# Regulation of micro-/nanotopography and porosity in biomimetic 3D-printed calcium phosphate ceramic scaffolds for enhanced bone regeneration

**DOI:** 10.1093/rb/rbag073

**Published:** 2026-04-17

**Authors:** Wenling Dai, Chenxin Liu, Shikui Li, Xingchen Zhao, Yumei Xiao, Changchun Zhou, Kate Jie Zhang Li, Likun Guo, Yujiang Fan, Xingdong Zhang

**Affiliations:** School of Biomedical Engineering, Sichuan University, Chengdu, Sichuan 610064, China; National Engineering Research Center for Biomaterials, Sichuan University, Chengdu, Sichuan 610064, China; School of Biomedical Engineering, Sichuan University, Chengdu, Sichuan 610064, China; National Engineering Research Center for Biomaterials, Sichuan University, Chengdu, Sichuan 610064, China; School of Biomedical Engineering, Sichuan University, Chengdu, Sichuan 610064, China; National Engineering Research Center for Biomaterials, Sichuan University, Chengdu, Sichuan 610064, China; School of Biomedical Engineering, Sichuan University, Chengdu, Sichuan 610064, China; National Engineering Research Center for Biomaterials, Sichuan University, Chengdu, Sichuan 610064, China; School of Biomedical Engineering, Sichuan University, Chengdu, Sichuan 610064, China; National Engineering Research Center for Biomaterials, Sichuan University, Chengdu, Sichuan 610064, China; School of Biomedical Engineering, Sichuan University, Chengdu, Sichuan 610064, China; National Engineering Research Center for Biomaterials, Sichuan University, Chengdu, Sichuan 610064, China; The Actions Company, Chengdu, Sichuan 641419, China; School of Biomedical Engineering, Sichuan University, Chengdu, Sichuan 610064, China; National Engineering Research Center for Biomaterials, Sichuan University, Chengdu, Sichuan 610064, China; School of Biomedical Engineering, Sichuan University, Chengdu, Sichuan 610064, China; National Engineering Research Center for Biomaterials, Sichuan University, Chengdu, Sichuan 610064, China; School of Biomedical Engineering, Sichuan University, Chengdu, Sichuan 610064, China; National Engineering Research Center for Biomaterials, Sichuan University, Chengdu, Sichuan 610064, China

**Keywords:** 3D printing technology, porous calcium phosphate ceramics, porosity, micro-/nanotopography, bone regeneration

## Abstract

Biphasic calcium phosphate (BCP) scaffolds were developed with the pivotal goal to biomimic natural bone tissue and enhance personalized and accurate repair to further fulfill the needs of regenerative medicine. 3D-printed BCP ceramics were extensively utilized in bone repair because of their customizable attributes and excellent biocompatibility. However, 3D printing technology and high-temperature sintering led to the absence of microporous structure and surface nanostructure in the scaffolds, which could hinder protein adsorption, osteogenic differentiation and bone regeneration. In this work, biomimetic BCP scaffolds featuring adjustable porosity and surface micro-/nanotopography were created by integrating 3D printing technology with a hydrothermal process. These BCP scaffolds had abundant micropores, and there were needlelike whiskers and hollow-tube whiskers distributed in them, which demonstrated special physical and biological properties. Compared with scaffolds sintered at high temperature, these BCP scaffolds possessed higher porosity and smaller grain size, thereby enhancing specific surface area (SSA), ions release, adsorption capacity of protein and facilitating osteogenic differentiation *in vitro*. In the rat cranial defect model, it was manifested that biomimetic BCP scaffolds could enhance *in situ* bone regeneration and showed significant osteoconductivity and osteoinductivity *in vivo*, which demonstrated their promise for deployment in bone tissue engineering and regenerative medicine.

## Graphical abstract

**Graphical Abstract:**
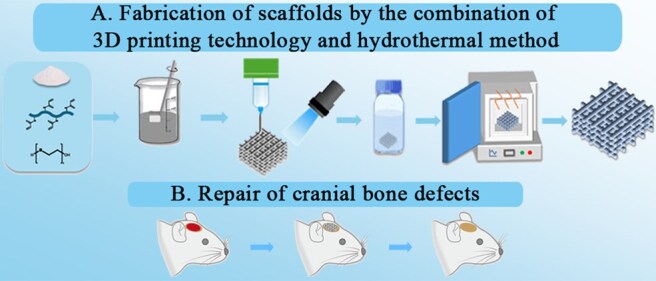

## Introduction

Natural bone tissue matrix was mainly composed of collagen and inorganic nanocrystallites, which could be considered as a nanocomposite material [1]. This nanocomposite material had a special hierarchical structure, where nanocrystallites (30–60 nm long, 1.5–10 nm thick) were present in the form of plates or needles, aligned parallel to the collagen fibers to create continuous mineral phase [1, 2]. These micro-/nanotopographies of natural bone were crucial for bone formation. Inspired by these, modification with special micro-/nanotopography on the scaffold was closer to the hierarchical features of natural bone, and therefore was widely used to regulate cell adhesion, migration, proliferation and differentiation and ultimately enhance bone regeneration *in vivo* [3–6]. It was worth noting that micro-/nanotopography on the scaffold surface could also regulate cell behavior in the absence of growth factors [7, 8]. In addition, micro-/nanotopography endowed the higher specific surface area (SSA) to biomimetic scaffolds, which would give additional adsorption sites to the bone-related proteins and activate the osteoblastic gene cascade [9, 10]. Therefore, integration of micro-/nanotopography on scaffolds would be facilitated to regulate cell behavior and enhance bone regeneration.

Calcium phosphate (CaP) ceramics were extensively utilized as scaffolds in tissue engineering to address the significant challenge of bone defect repair. Their composition closely resembled that of natural bone tissue, and they possessed excellent biocompatibility, osteoconductivity and osteoinductivity [11, 12]. Currently, biphasic calcium phosphate (BCP) ceramics have gained considerable attention as bone regeneration scaffolds by reason that their prominent osteoinductivity and suitable solubility [13, 14]. Nevertheless, the bioactivity of traditional BCP scaffolds was still inferior to that of natural bone tissue. It might be related to the high crystallinity of the ceramics after high-temperature sintering and insufficient nanoscale structures on their surface [15]. The crystal grew with the rising temperature, which ultimately resulted in a larger grain size of BCP and affected the porous structure of the scaffolds [16]. In contrast, calcium-deficient hydroxyapatite (CDHA) had a lower crystallinity and a chemical composition and crystal structure closer to natural bone minerals, thereby exhibiting better bioactivity [17, 18]. It was reported that the method, which stabilized the CDHA phase in BCP ceramics, could prepare scaffolds to mimic natural bone minerals, and the osteoinductivity of BCP scaffolds with CDHA phase were improved *in vivo* [19, 20]. Obviously, the introduction of CDHA phase into BCP scaffolds could enhance osteogenic activity and bone regeneration.

With the progress in technology and processes, additive manufacturing technologies have created applications in the bone repair field. It could be personalized by 3D printing technology to prepare scaffolds with complex structures and repair irregular bone defects. It was an urgent problem that 3D printing scaffolds lacked rough surfaces and micropores on the walls to support cell growth and differentiation [9, 21, 22]. The photolithography, template method and other methods were used to fabricate micro-/nanotopography on porous bioceramics, but these methods showed high cost, complex processes and trouble of extending the 2D pattern to the 3D scaffolds [21, 23]. The preparation methods of micro-/nanotopography of bioceramics were mostly focused on the hydrothermal method [4, 24–27]. In a high-pressure water vapor environment, the supersaturated Ca^2+^ and ${\mathrm{PO}}_{4}^{3-}$ caused recrystallization on the bioceramic surface, forming crystals and constructing a micro-/nanotopography [28, 29], and micro-/nanotopography on the scaffolds could regulate cell behavior and be essential for bone tissue regeneration.

**Figure 1:**
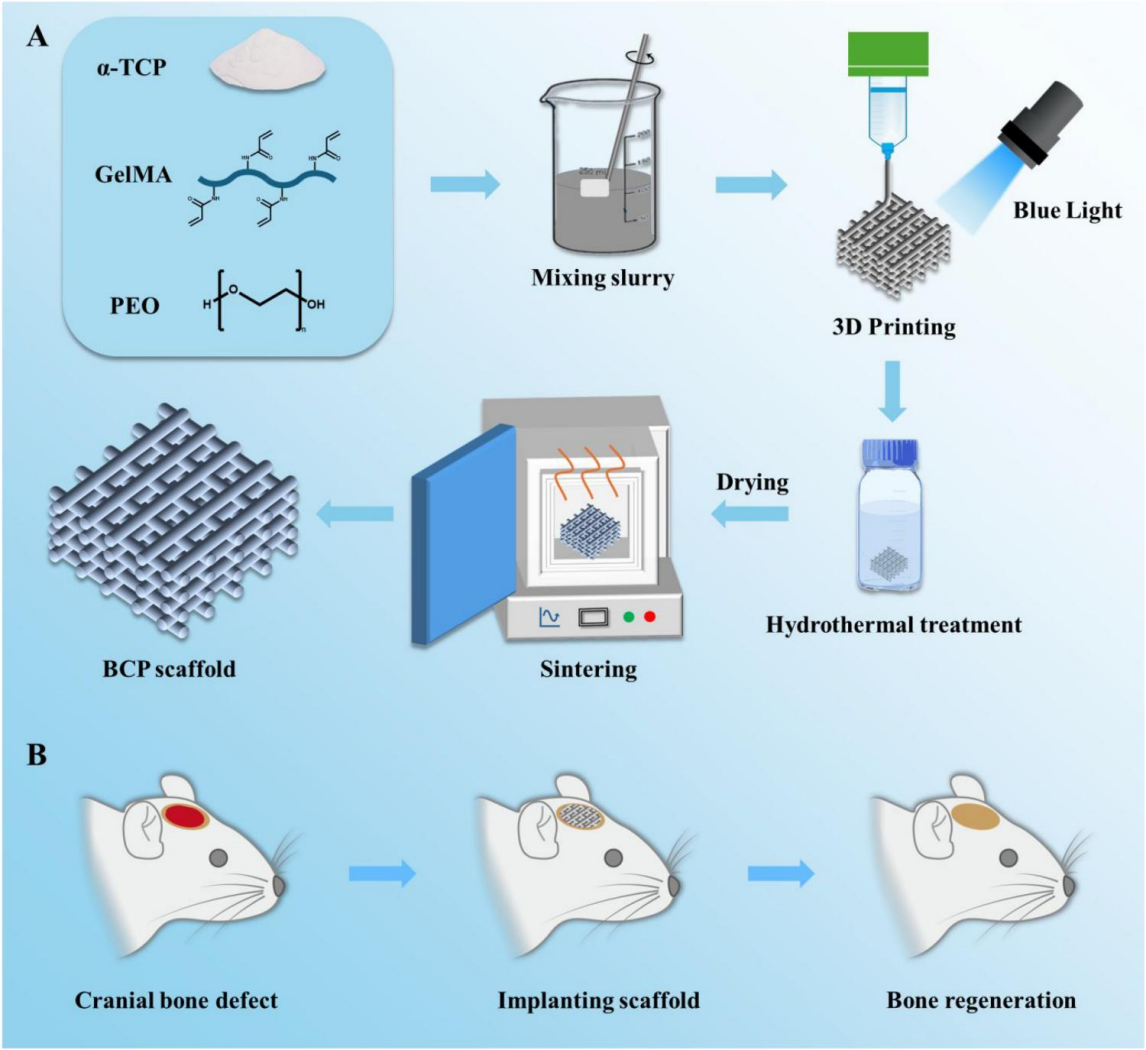
(**A**) Schematic diagram for preparation of BCP scaffolds. (**B**) Schematic illustration on treatment of cranial bone defects.

In this work, the biomimetic BCP scaffolds were constructed by integrating 3D printing technology with a hydrothermal process (Figure 1). Obtained BCP scaffolds with different surface micro-/nanotopography and adjustable porosity were performed for bone regeneration. The micro-/nanotopography was fabricated on the BCP scaffolds while the hydrolysis of α-tricalcium phosphate (α-TCP) to CDHA was accelerated through the hydrothermal approach. After sintering, the BCP scaffolds with different surface micro-/nanotopography and different porosity were obtained. Their surface morphologies, porous structures and other properties were characterized. Moreover, the effects of different scaffolds on protein adsorption, cytocompatibility and osteoblastic differentiation of bone marrow-derived mesenchymal stem cells (BMSCs) were studied *in vitro*. Finally, the biomimetic BCP scaffolds were implanted in rat cranial bone defects to verify the bone repair effect *in vivo*.

## Materials and methods

### Materials

α-TCP powder was provided by the National Engineering Research Center for Biomaterials at Sichuan University. Methacrylated gelatin (GelMA) was synthesized in our laboratory by grafting methacrylate onto gelatin. Lithium phenyl-2,4,6-trimethylbenzoylphosphinate (LAP) was supplied by SinoBioPrint Biotech Ltd. (Shanghai, China). Polyethylene oxide (PEO) was supplied by Hefei BASF Biotechnology (Hefei, China). Sodium dodecyl sulfate (SDS) was provided by Thermo Fisher Scientific. BCA protein assay kit and alkaline phosphatase (ALP) assay kit were purchased from Beyotime Biotechnology (China). Mouse-derived BMSCs were provided by Cyagen Biotechnology Inc. (Suzhou, China).

### Fabrication of BCP ceramic scaffolds

Firstly, 10 wt%, 20 wt%, 30 wt%, 50 wt% GelMA solutions were prepared in 2 mg mL^−1^ LAP solution, respectively. Then, the 3D printing inks were prepared by mixing α-TCP, 15 wt% PEO solution and different GelMA solutions in the ratio of 4:1:1. Eventually, these inks were placed in a defoaming apparatus (THINKY, ARE-310, Japan) to remove the foam and utilized to fabricate scaffolds.

Modeling software was used to create the 3D printing model, which measured 10 × 10 × 2 mm for each sample. The material-extrusion-based 3D printer (Regenovo, China) with a 400 μm diameter nozzle was used to produce the printed green body. They were printed layer by layer at a speed of 8 mm s^−1^. The layer height was set to 0.32 mm. The printed green body was cross-linked by blue light to provide incipient mechanical properties of green body. Then these green bodies were immersed in water and processed at 120°C and 0.1 atm for 30 min. Finally, these green bodies were sintered at 800°C for 2 h. According to the concentration of the added GelMA, these scaffolds were designated as BCP-10, BCP-20, BCP-30 and BCP-50. The green bodies containing 20% GelMA were sintered to 1100°C and treated as a control group.

### Characterization of BCP ceramic scaffolds

The phase compositions of the BCP ceramic scaffolds were determined by X-ray diffractometry (XRD), and then, based on the obtained XRD results, the lattice parameters and crystal sizes of different phase compositions of obtained scaffolds were calculated using Jade 6.0 software. The surface topography of the BCP scaffolds were observed by the scanning electron microscope (SEM, S4800, Hitachi, Japan). Nitrogen adsorption method was used to calculate the SSA of BCP scaffolds using the Brunauer–Emmett–Teller technique (Micromeritics, USA). The mechanical strengths of these BCP scaffolds were investigated using a universal testing machine (AGS-X, Shimadzu, Japan).

### Porosity analysis

According to the formerly reported method [18], the porosity of different BCP scaffolds was determined using a drainage method. In brief, *W*_1_ denoted the weight of scaffolds; *W*_2_ denoted the apparent weight of the scaffolds after soaking; *W*_3_ denoted the measured weight of the scaffolds immersing in water. The porosity was calculated as follows:


$$\mathrm{Porosity}\;(\%)=\frac{W_{2}-W_{1}}{W_{2}-W_{3}}\times 100\%$$

### Protein adsorption

The samples were soaked with 2 mg mL^−1^ bovine serum albumin (BSA) at 37°C for 4 h, after that washed and placed in the 2% SDS solution for 4 h. The protein adsorption was quantified using BCA protein assay kit.

### Ion release test

The different scaffolds were incubated with α-minimum essential medium (α-MEM) at 37°C. At 0, 1, 4 and 7 days, samples were collected and analyzed to determine the concentrations of Ca^2+^ using an inductively coupled plasma optical emission spectrometer (ICP-OES, 7900, Agilent, USA).

### Cytocompatibility and cell spreading

BMSCs were utilized for cell experiments *in vitro*. The scaffold was immersed in PBS and serum-free medium for 24 h. BMSCs were cultured with α-MEM containing 10% fetal bovine serum. BMSCs were seeded on the BCP ceramics scaffolds with a density of 7 × 10^4^ cells per sample. After culturing, cells on ceramic scaffolds were stained with fluorescein diacetate and propidium iodide. They were then viewed using a confocal laser scanning microscope (CLSM, TCS-SP5, Leica, Germany). Cell proliferation was measured by cell counting kit-8 (CCK-8) reagent. Additionally, the sample with cells were fixed with 4% paraformaldehyde (PFA), dehydrated, dried by a critical point dryer (CPD300, Leica, Germany) and then observed by SEM.

### ALP activity assay

After culturing for 7 and 14 days, the samples were harvested, and the intracellular ALP activity was colorimetrically tested using assay kit.

### Rat cranial bone defects repair

All animal studies in this work were approved by the Animal Care and Use Committee of Sichuan University (No. K2023014). *In vivo* studies were conducted using rats (male, 180–220 g). Cylindrical full-thickness defects with a diameter of 5 mm were created on the calvaria of anesthetized rats using dental trephine. The scaffold was transplanted into defect. After 8 weeks, the samples were collected and fixed in 4% PFA for subsequent investigation.

### Micro-computed tomography analysis and histological analysis

Micro-computed tomography (Micro-CT; Scanco Medical AG, Switzerland) was used to examine the new bone formation in samples after implantation. The voltage was set at 70 kV, and the resolution of 15 μm. Then, the samples were decalcified using 10% EDTA solution (pH 7.4). Finally, the samples after dehydration were prepared as paraffin sections, which were used for hematoxylin and eosin (H&E) and Masson’s trichrome staining.

### Statistical analysis

The quantitative data in this work were collected from at least triplicate measurements (*n* ≥ 3) and expressed as mean ± SD. A one-way analysis of variance was used to determine statistical significance across groups. *P *< 0.05 was considered to be significant; *P < 0.05, **P < 0.01, ***P < 0.001 and ****P < 0.0001.

## Results and discussion

### Phase composition and morphology

**Figure 2:**
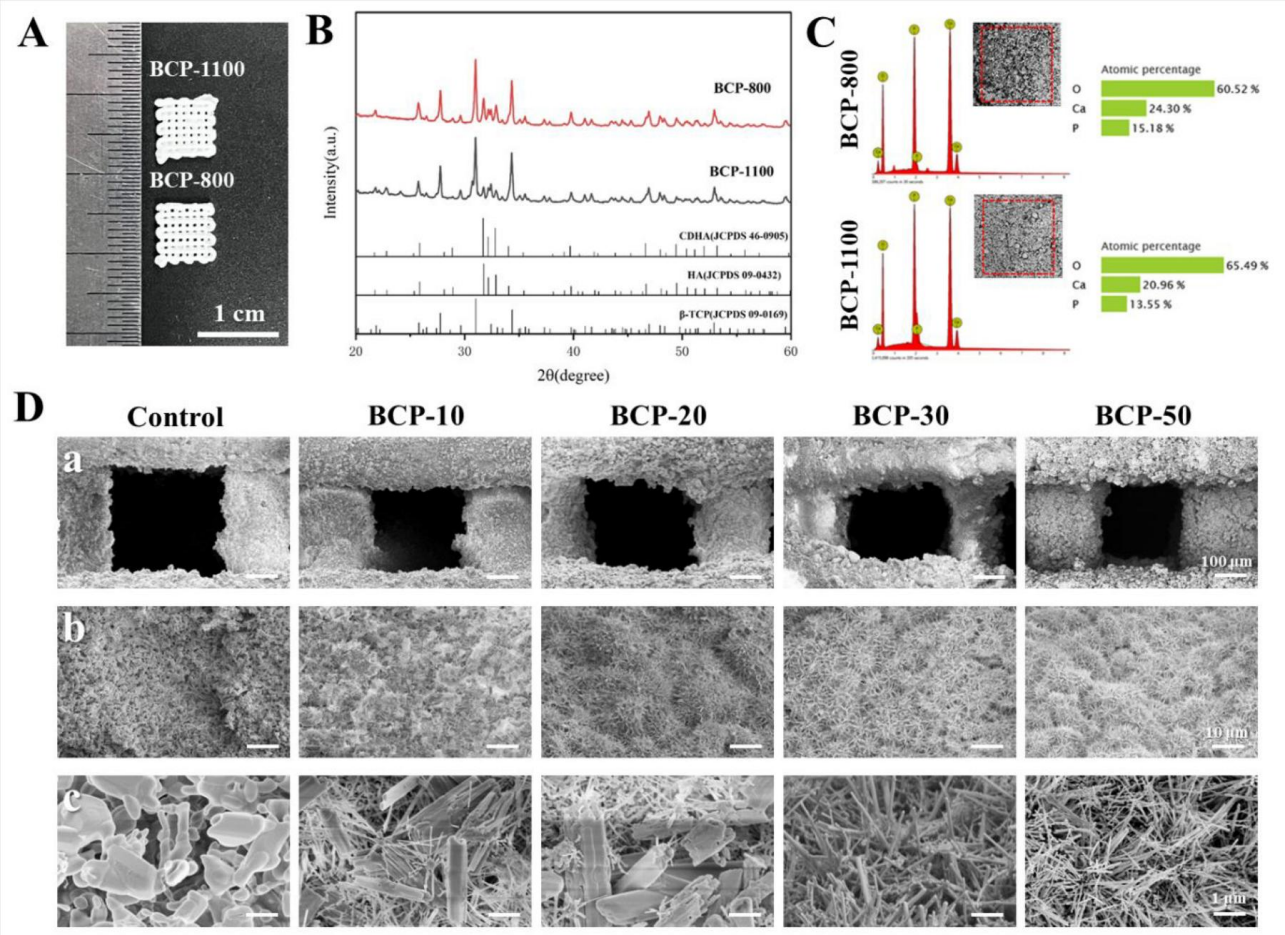
Characterization of BCP scaffolds. (**A**) Photograph of 3D-printed BCP scaffolds after sintering at different temperatures. (**B**) XRD patterns of 3D-printed BCP scaffolds sintered with different temperatures. (**C**) SEM images and EDS spectrum of BCP scaffolds sintered with different temperatures. (**D**) Surface morphology of 3D-printed BCP scaffolds.

The sintering temperature played a pivotal role in the preparation of BCP scaffolds as it significantly impacted their phase composition, physical properties and surface microstructure variation [11, 30]. To investigate the impact of sintering temperature on the scaffolds, 3D printed green bodies containing 20% GelMA were subjected to sintering at 800 and 1100°C, and they were designated as BCP-800 and BCP-1100. The BCP scaffolds were exhibited in Figure 2A. XRD patterns of scaffolds were compared in Figure 2B, and the standard references for CDHA, HA and β-TCP were used to identify the acquired diffraction peaks. The phase compositions of BCP-800 and BCP-1100 included β-TCP and HA. By calculating, as the sintering temperature rose, the content of the HA phase in the scaffolds reduced (Table 1). Moreover, BCP-800 had smaller grain size and lower crystallinity than BCP-1100. Figure 2C depicted the calcium–phosphorus ratio in different scaffolds as measured by an energy dispersive spectrometer (EDS). BCP-800 had a greater calcium to phosphorus ratio (1.60) than BCP-1100 (1.55). The α-TCP phase in the green body was converted into CDHA phase, which had unstable crystalline and low crystallinity, during the hydrothermal reaction. After sintering at 800 and 1100°C, unstable CDHA underwent thermal decomposition, producing β-TCP and HA. At a sintering temperature of 800°C, some CDHA was present in the BCP-800 scaffold, however, CDHA would be completely transformed into β-TCP once the sintering temperature increased to 1100°C [18, 31, 32]. It was well recognized that CDHA resembled natural bone minerals better in terms of chemical constituent and crystal structure than other calcium–phosphate materials [33, 34]. The introduction of CDHA into scaffolds could not only enhance their biological performances but also better imitate the inorganic composition of real bones from the perspective of biomimetics [19, 35]. Furthermore, increasing temperatures might accelerate molecular movement, which could enhance densification and the grain size development that occurred in the scaffolds [36]. This would result in a significantly smaller grain size and lower crystallinity of BCP-800 compared to BCP-1100.

**Table 1:** The phase ratios, lattice parameters and crystal sizes of different scaffolds.^a^

	Phase	Ratio (%)	*a* = *b* (nm)	*c* (nm)	*V* (nm^3^)	XS (nm)	Crystallinity (%)
BCP-800	HA	28.6	0.9462	0.6849	0.5310	42.6	79.4
	β-TCP	71.4	1.0439	3.7375	3.5271		
BCP-1100	HA	19.4	0.9424	0.6879	0.5291	53.6	89.6
	β-TCP	80.6	1.0462	3.7471	3.5517		

a XS represented the crystal size determined using either the HA main peak (211) or the β-TCP main peak (0210).

The surface morphologies of scaffolds were shown in Figure 2D. The scaffolds had regular orthogonal macropores with a size of approximately 300 μm. Those macropores served as constructed channels for substance exchange were beneficial to guarantee a sufficient supply of nutrients, encouraging quick bone ingrowth and vascularization [37, 38]. Due to higher temperatures, scaffolds in the control group exhibited larger grain sizes and a denser microstructure, resulting in a lack of micropores. By contrast, scaffolds in the other groups exhibited abundant micropores and entangled network of nanocrystals and varied micro-/nanotopographies. The nanoscale needlelike whiskers and hollow-tube whiskers were found on BCP-10 and BCP-20. There were nanoscale needlelike whiskers observed on BCP-30 and BCP-50. During hydrothermal treatment, Ca^2+^ and ${\mathrm{PO}}_{4}^{3-}$ from surface erosion of α-TCP in the scaffolds were continuously released and reached supersaturation, which could result in the hydroxyapatite nucleated on the eroded surface [39]. However, the competing growth of crystal might have been affected by GelMA content, thereby resulting in different micro-/nanomorphologies on the scaffolds [40, 41]. In this study, the BCP scaffolds were fabricated by hydrolyzing α-TCP to CDHA, and then thermal decomposition of CDHA at low temperatures. Changing the GelMA content in the slurry could adjust the shape and size of the crystals, consequently modifying the micro-/nanomorphologies of the scaffolds. While the crystals of BCP scaffolds were still slightly larger than those of natural bone minerals, this method made it possible to approach biological apatite [42]. The smaller grain size, abundant micropores could conduce to the increase of SSA and surface roughness, which further could positively affect biological performances of BCP scaffolds [43, 44].

### Porosity and SSA

**Figure 3:**
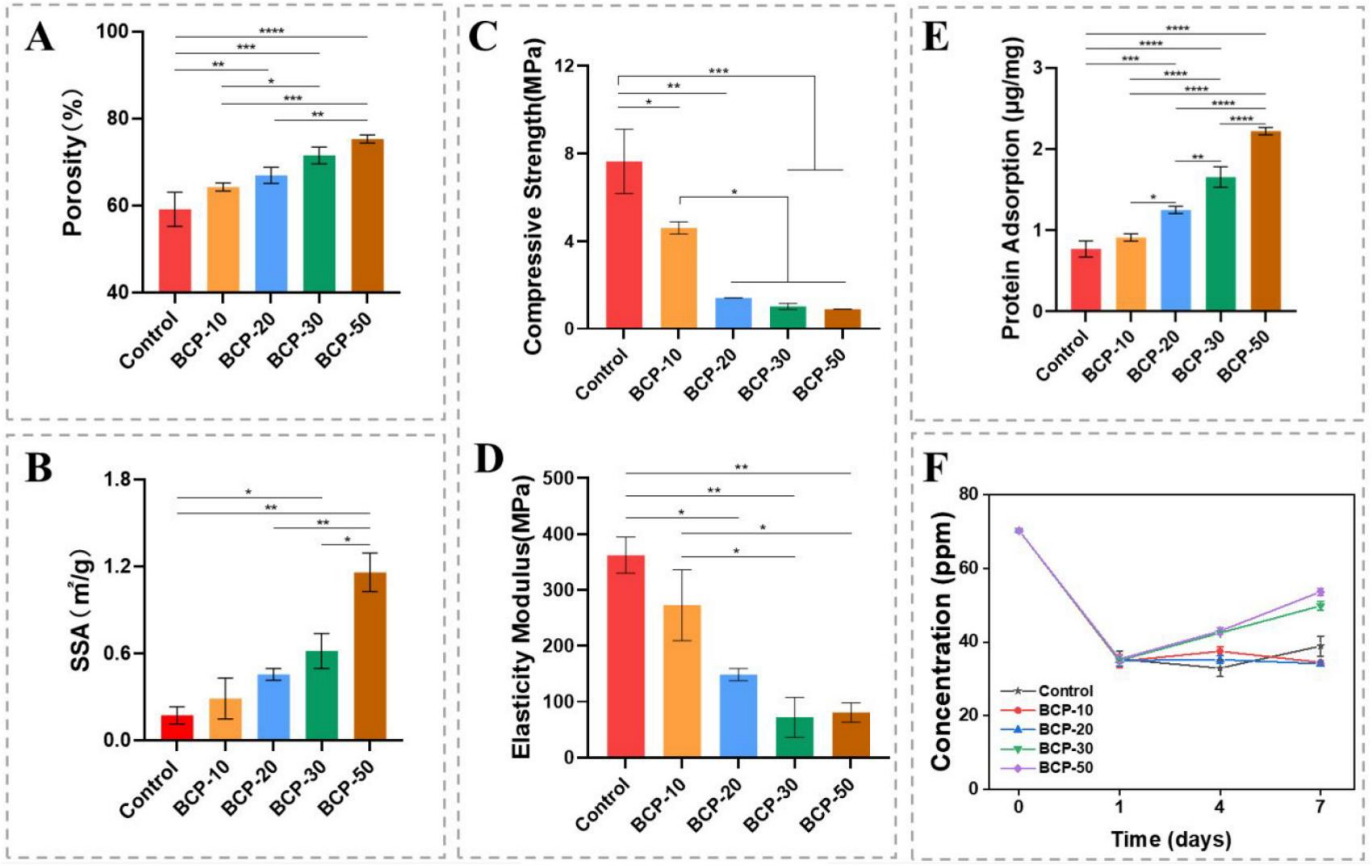
(**A**) Porosity of different BCP scaffolds. (**B**) Specific surface area of different BCP scaffolds. (**C**) Compressive strength and (**D**) elasticity modulus of different BCP scaffolds. (**E**) Protein adsorption of different BCP scaffolds. (**F**) Calcium ion release curve of different BCP scaffolds. *P < 0.05, **P < 0.01, ***P < 0.001 and ****P < 0.0001.

In Figure 3A, the porosity of control, BCP-10, BCP-20, BCP-30 and BCP-50 scaffolds respectively was 59.10 ± 4.55%, 64.25 ± 1.05%, 66.93 ± 1.94%, 71.50 ± 2.06% and 75.28 ± 1.01%. These results indicated that increasing the concentration of GelMA in the initial slurry improved the porosity of BCP scaffolds. Meanwhile, the SSA of BCP scaffolds also increased with the increase of GelMA concentration (Figure 3B). The SSA of BCP-50 was the highest, reaching 1.16 ± 0.09 m2 g−1. Additionally, porosity and SSA of BCP scaffolds sintered at 1100°C were lower than those of BCP scaffolds sintered at 800°C. This change occurred due to enhanced densification and grain size of the scaffolds at high temperatures. In this study, GelMA could not only perform photo-crosslinking to solidify the green body of scaffolds but also affect the whisker growth and space occupation to regulate micro-/nanomorphology and porosity of scaffolds, also further affecting SSA. Scaffolds characterized by high porosity and SSA were crucial for bone tissue engineering [45]. For bioactive ceramic scaffolds, porosity levels between 40% and 80% are suitable for repairing bone tissue [46]. The interconnected scaffolds with abundant micropores could facilitate protein adsorption and stimulate osteoinduction occurrence in vivo [47]. Furthermore, the SSA of the BCP scaffold was positively correlated with the concentration of GelMA, which benefited from the gradually tiny micro-/nanomorphology. The high SSA and nanoscale surface topography could efficiently regulate a cascade of cellular activities, thereby enhancing the osteogenic environment and facilitating osteogenic differentiation [4, 8, 44].

### Mechanical property

The compressive strength and elasticity modulus of BCP scaffolds were presented in Figure 3C and D, respectively. These data correlated with the porosity, and both parameters decreased as the porosity increased. The causes were directly related to the sintering temperature and GelMA content of scaffold [27]. The mechanical properties of the scaffolds were able to satisfy the cancellous bone, rendering them suitable for applications in non-weight-bearing bone repair, e.g., the cranial bone and maxillofacial bone. Perhaps they could leverage the advantages of 3D printing to improve the mechanical support of these scaffolds by regulating the pore structure, thereby enabling their application in a wider range of bone tissue repair applications [48, 49].

### Protein adsorption

The first trigger occurrence which occurred at the interface between biomaterials and tissue was protein adsorption. BCP scaffolds were submerged in BSA solution to test their capacity to adsorb proteins. The results indicated that the protein adsorbing ability of scaffolds from high to low was in the following order: BCP-50 > BCP-30 > BCP-20 > BCP-10 > control scaffold (Figure 3E). It was well known that protein adsorption was an extremely complex process, which was influenced by surface topography, porosity, SSA and other characteristics of biomaterials [50]. The above BCP scaffolds had different micro-/nanomorphology, and nanoneedle-like and hollow-tube whiskers on the BCP scaffolds created numerous active sites for protein adsorption [4, 27]. Compared to other scaffolds, BCP-50 scaffolds had more micropores and a bigger SSA, which might provide more active sites for protein adsorption. The proteins were firmly adsorbed on the surface of BCP scaffolds by electrostatic interaction as the main driving force [51]. Meanwhile, these proteins could provide a temporary matrix to promote cell adhesion and influence osteogenic differentiation [52, 53].

### Calcium ion release

As shown in Figure 3F, the Ca^2+^ concentration variation of each BCP scaffold in α-MEM could be monitored. The initial Ca^2+^ concentration of α-MEM was 70.3 ppm. At 1 day, there was a significant decrease in the Ca^2+^ concentration after adding the scaffolds, which meant that the scaffolds had begun to absorb Ca^2+^ from the medium. The Ca^2+^ concentration in the medium subsequently increased at 4 days. Simultaneously, Ca^2+^ release of BCP-30 and BCP-50 exceeded that of other scaffolds, and the trend continued for 7 days. Higher SSA might have accelerated ions release from scaffolds, suggesting that BCP-30 and BCP-50 exhibited greater activity than other scaffolds [54]. Generally, Ca^2+^ was recognized as key factors in the osteoinductive phenomenon in CaP ceramics [11]. For example, Ca^2+^ might initiate the process of bone induction by influencing the adsorption of proteins [50]. Moreover, the Ca^2+^ could not only be used as a homing signal to improve cell migration but also encourage cell proliferation and differentiation to drive bone regeneration [55].

### Cell proliferation, morphology and ALP activity

**Figure 4:**
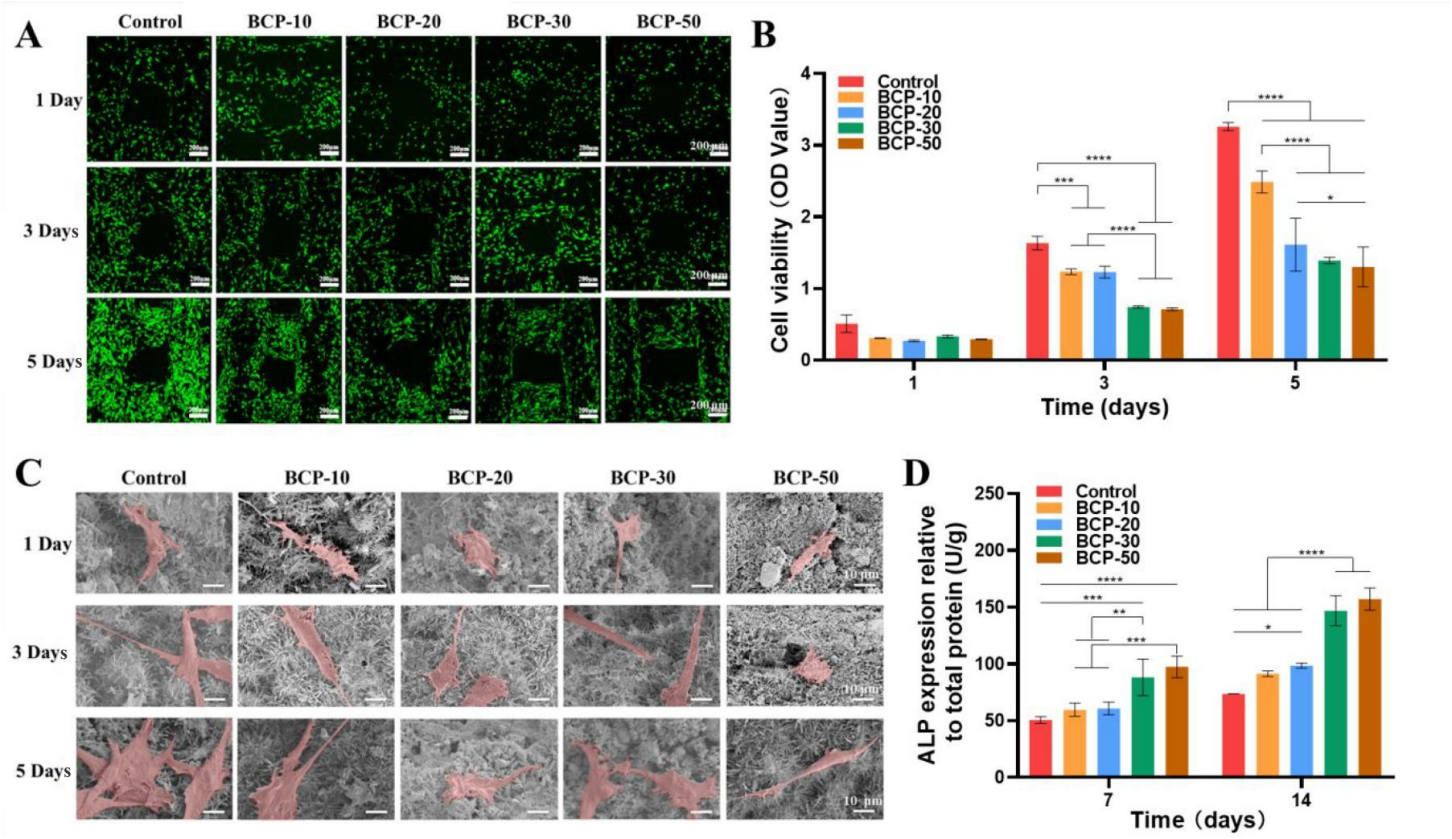
*In vitro* biological performance of 3D-printed BCP scaffolds. (**A**) CLSM observation of live/dead staining, (**B**) CCK-8 results and (**C**) SEM observation of BMSCs grown on different BCP scaffolds. (**D**) ALP protein expression in BMSCs cultured in different scaffolds. *P < 0.05, **P < 0.01, ***P < 0.001 and ****P < 0.0001.

The growth of BMSCs on the scaffold could be visually observed by live/dead staining (Figure 4A). The results indicated that there were hardly any dead cells and the number of BMSCs on each scaffold increased after 5 days of culture. The cell proliferation could be quantitatively assessed by CCK-8 (Figure 4B). BMSCs on each scaffold proliferated obviously. However, the cell proliferation rate of BCP groups except the control was slow, which might be attributed to their nanomorphology. The spreading of BMSCs on the scaffold surface was observed (Figure 4C). BMSCs adhered closely and spread well on the control and BCP-10 groups after culture. In the BCP-20 and BCP-30 groups, the area of BMSCs spreading significantly increased after culturing for 3 days. After 5 days of culture, BMSCs of the BCP-50 group also showed well spreading. At this point, BMSCs on all the groups exhibited a spindle-like morphology. Such a phenomenon had been observed, which was numerous filopodia of cells projecting to tightly clasp the ceramic grains or whiskers. ALP was a crucial prophet of osteogenic differentiation as well as a particular inducer of bone regeneration [56]. ALP activity in BMSCs cultured on BCP scaffolds was quantitatively detected (Figure 4D). Surprisingly, BMSCs seeded on BCP-30 and BCP-50 scaffolds exhibited significantly elevated ALP activity at 7 and 14 days.

The surface topography of scaffolds had a profound impact on cellular behaviors. The previous study certified that the bioactive sites in the scaffolds might increase after hydrothermal treatment, which could be conducive to biological performances of BCP ceramics [24]. In this study, BCP scaffolds with whiskers could supply a microenvironment for cell adhesion and spreading. But the scaffolds with different surface topography showed significant differences for cell proliferation and osteogenic differentiation, which was consistent with previous reports [54, 57]. The scaffolds of the control group were more supportive for cell proliferation than the other groups. BCP scaffolds with whiskers were conducive to protein absorption, but the nanoscale needlelike whiskers were sharp, which was sharp for BMSCs and could also ‘stab’ the cell to death [4]. However, the BCP scaffolds with whiskers showed surprising potential for osteogenic differentiation, especially for BCP-30 and BCP-50 scaffolds. It was confirmed by the previous study that nanoscale whiskers could facilitate osteogenesis, which might be the result of a combination of factors such as SSA, ions release, porosity and others [58–61]. Therefore, further studies *in vivo* were warranted to evaluate the osteogenic effect of the scaffold.

### *In situ* bone repair

**Figure 5:**
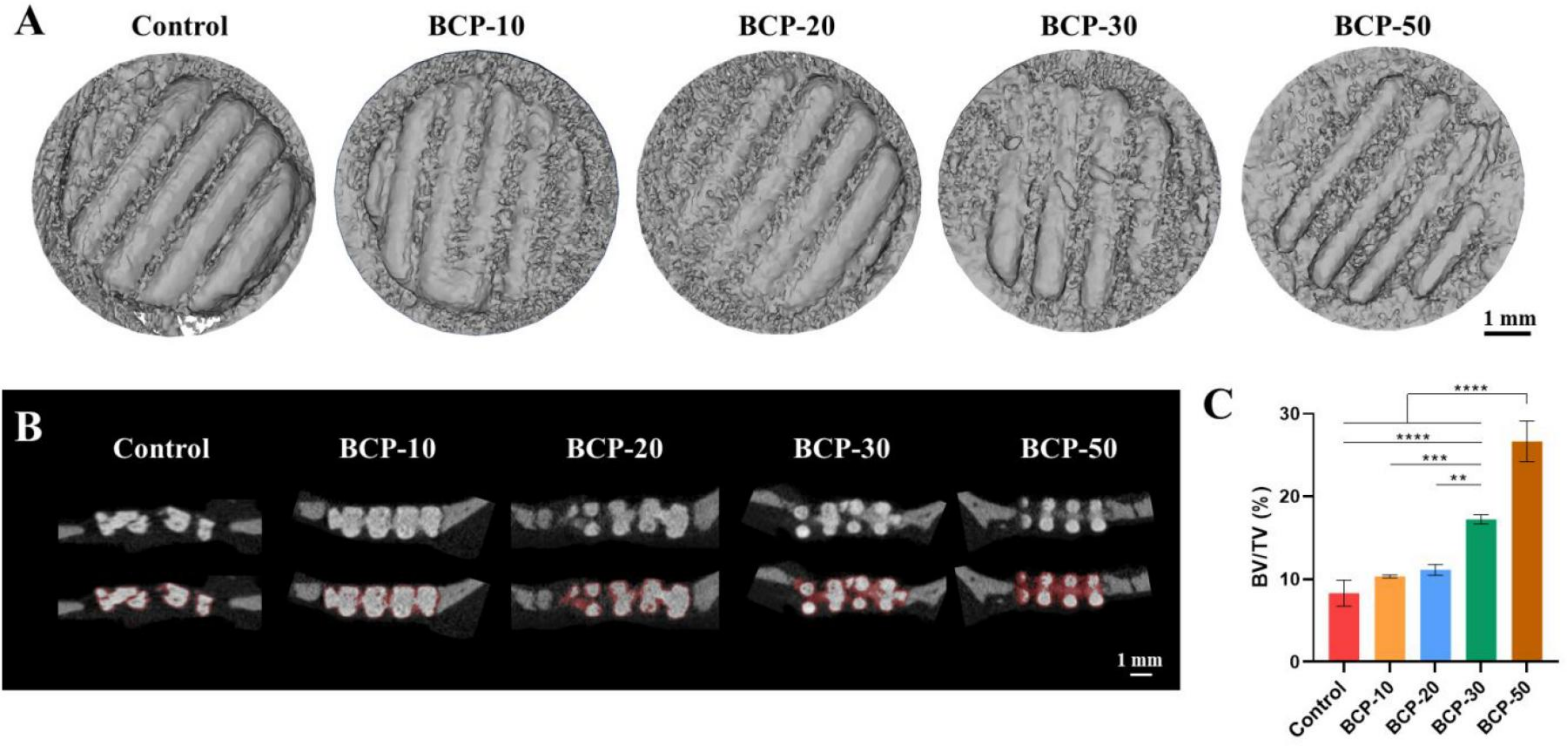
New bone formation in bone defects treated with different BCP scaffolds. (**A**) Reconstructed micro-CT images after 8 weeks. (**B**) Micro-CT analysis of cranial bone defects with side view (white part denotes scaffold; red part denotes new bone). (**C**) Quantification of bone volume fraction after 8 weeks. **P < 0.01, ***P < 0.001 and ****P < 0.0001.

The rat cranial bone defect model was developed to assess the *in situ* bone repair capabilities of BCP scaffolds. The new bone formation of the scaffolds was assessed by micro-CT reconstruction. As shown in Figure 5A, the scaffolds in the BCP groups showed obscured contours and had a certain degree of degradation. Their defect gaps were filled with confluent bones. However, a small part of defect gaps was still present in the control group. The repair tissue of BCP-30 and BCP-50 scaffolds showed no clear boundary with the host bone and more uniform integration with the surrounding normal bone. Further investigation indicated that the distribution of new bone varied among the groups from the sectional view (Figure 5B). The new bone was mainly distributed at the edge of the scaffold in the control, BCP-10 and BCP-20 groups. In the BCP-30 and BCP-50 groups, new bones mainly developed near the scaffold’s border and within the interconnected macropores that were distant from the host bone, and numerous new bones were discovered. Quantification of micro-CT analysis revealed that the bone volume fraction (BV/TV) followed the order of control < BCP-10 < BCP-20 < BCP-30 < BCP-50 after 8 weeks post-implantation (Figure 5C). The BV/TV of BCP-30 and BCP-50 scaffolds were higher than others with a significant difference.

**Figure 6:**
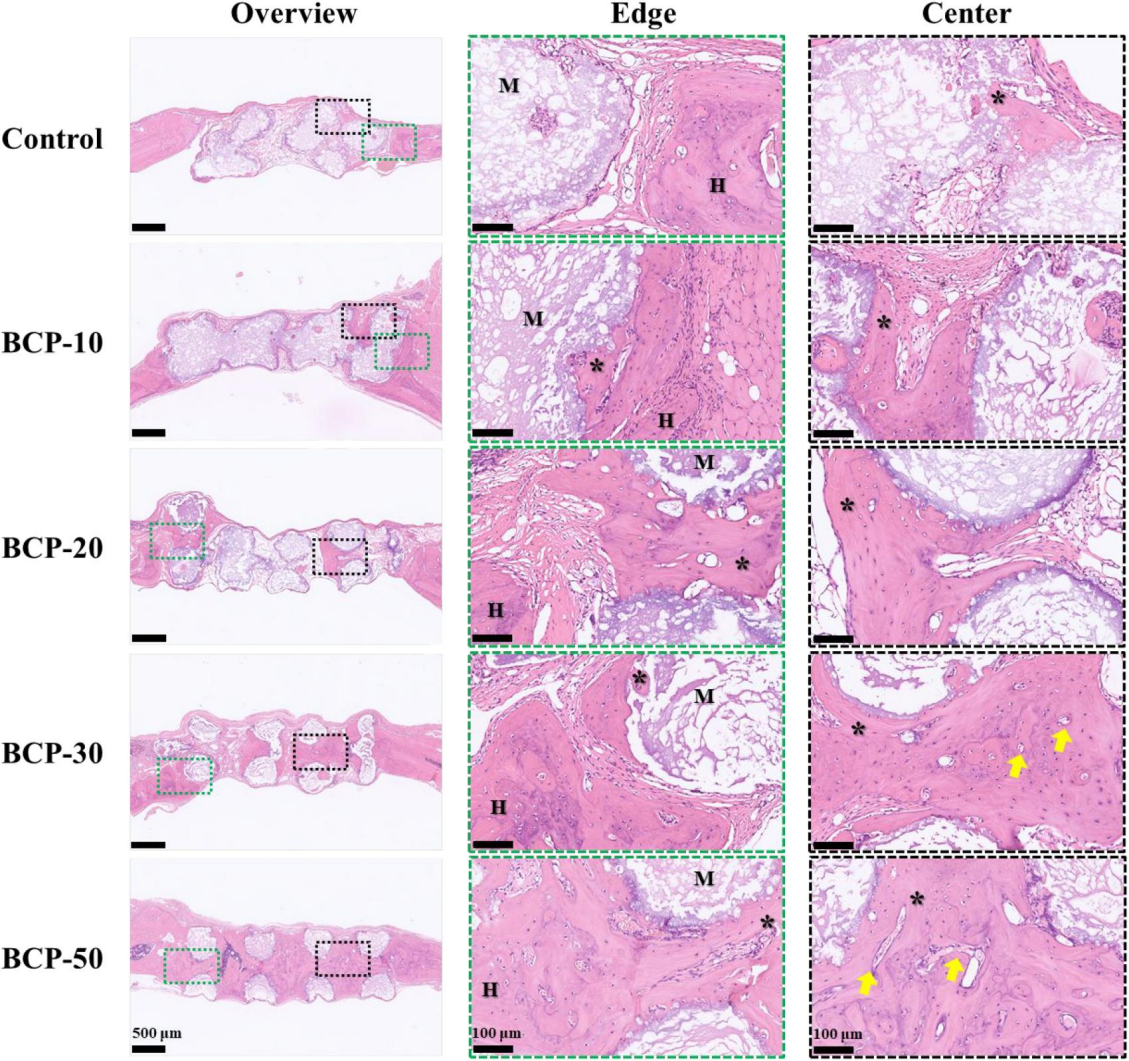
H&E staining evaluation of *in vivo* bone regeneration. The dashed green box shows the repair situation of the junction between normal and repaired tissue. The dashed black box shows the regeneration situation of new tissue in the scaffold. (M indicates material, H indicates host tissue, * indicates new bone, the yellow arrows indicates new capillary vessel).

**Figure 7:**
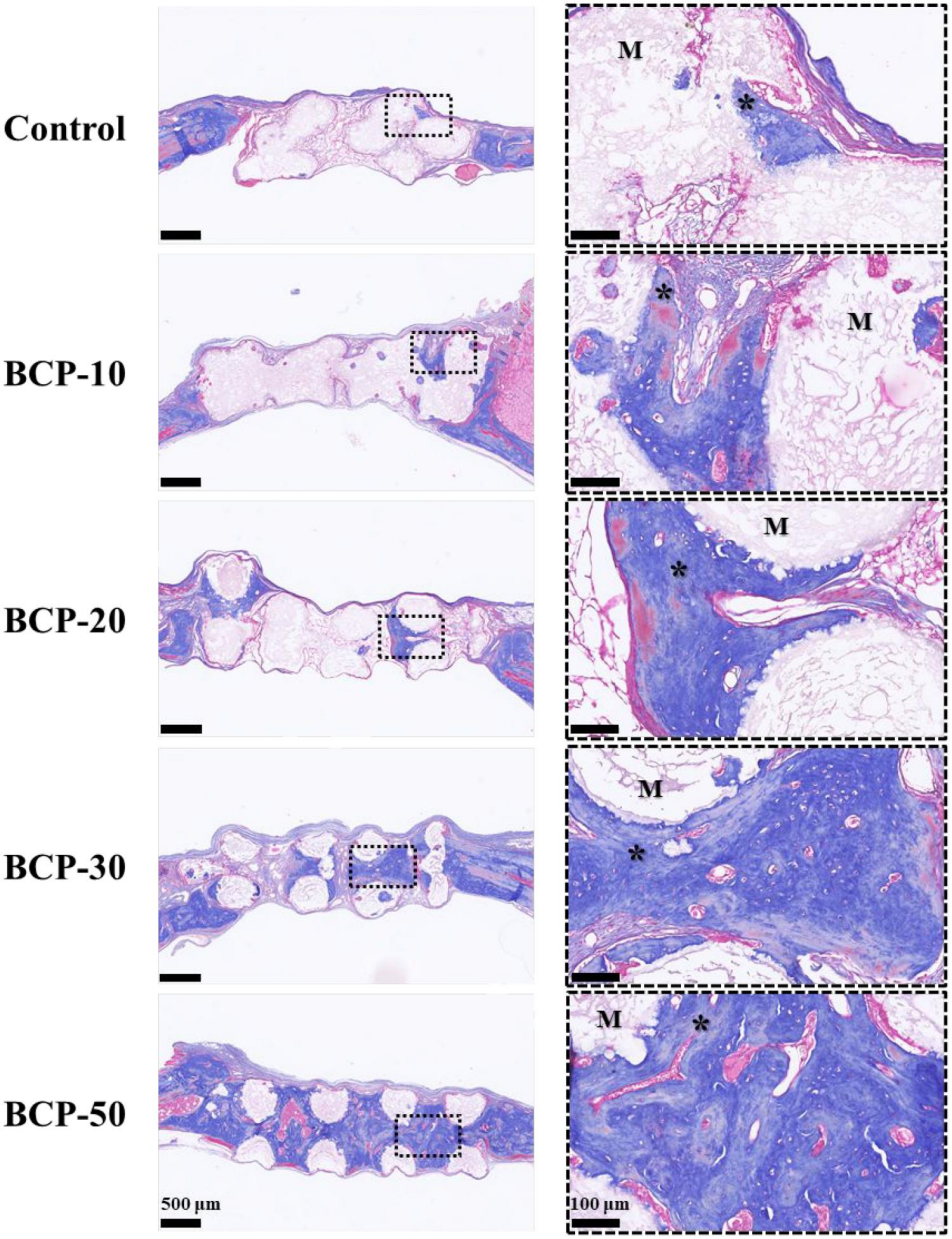
Masson’s trichrome staining evaluation of *in vivo* bone regeneration. The dashed black box shows the regeneration situation of new tissue in the scaffold. (M indicates material; * indicates new bone).

The results of H&E staining were shown in Figure 6. At 8 weeks, the new bone developed alone edge of the BCP scaffolds near the host bone except the control group. Furthermore, even in regions distant from the natural bone, the new bone tissue was discovered inside the interconnecting macropores of every scaffold. The BCP-30 and BCP-50 groups showed a considerable increase in both the number of blood vessels and the areas of new bone formation. The Masson staining images showed comparable results (Figure 7). It demonstrated that plentiful collagen matrix and observably more mature bone tissue generated in the BCP-30 and BCP-50 groups. The results suggested that BCP-30 and BCP-50 scaffolds exhibited robust osteoconductivity and osteoinductivity.

After the scaffold was implanted *in vivo*, its surface directly contacted host bone tissues. The micro-/nanomorphologies of the scaffold surface would be crucial for controlling cellular responses, which also encouraged extensive investigation into how the scaffold surface affected biological responses [62, 63]. Therefore, enhancing scaffolds with improved biological activity to meet the requirements of bone repair and regeneration had become an eternal topic when designing biomaterials. In this study, the BCP scaffolds were produced by combining 3D printing technology with the hydrothermal method, and their micro-/nanomorphology and porosity were regulated based on GelMA content in the slurry. Simultaneously, SSA and other characteristics were also controlled during scaffolds preparation. These characteristics of BCP scaffolds provided the prerequisite for bone regeneration and vascularization in the BCP-30 and BCP-50 scaffolds with nanoscale needlelike whiskers and high porosity. The nanoscale whiskers on the scaffold surface could mimic the structure of the natural bone extracellular matrix, thereby possessing the ability to control the signaling cascades that resulted in accelerating osteogenic differentiation of stem cells and encouraging angiogenesis [3, 6, 41]. The high porosity could endow interconnected scaffolds with the capacity to enhance bone ingrowth and bone regeneration [64]. More importantly, the micro-/nanomorphologies and porosity could contribute to enhancing SSA, release of ions and protein adsorption, which led to earlier cell mineralization and improved osteoconductivity of scaffolds [9, 12, 64]. In consequence, the scaffolds with nanoscale needlelike whiskers and high porosity not only could mimic natural bone minerals but also had high SSA and high capability of protein adsorption, which would build a microenvironment to support osteogenic differentiation, promote bone ingrowth and guide bone regeneration efficaciously.

## Conclusion

In this study, the biomimetic BCP scaffolds with adjustable surface micro-/nanotopography and porosity were developed by combining 3D printing technology with the hydrothermal process. These scaffolds possessed abundant micropores and different surface micro-/nanotopography that could be regulated by GelMA concentration. With the increase of GelMA concentration, the porosity of scaffolds increased, and grain size of scaffolds decreased, which could enhance SSA, ions release and protein adsorption, thereby promote osteogenic differentiation. The results of rat cranial bone defects repair demonstrated that the biomimetic BCP scaffolds possessed the capacity of enhancing *in situ* bone regeneration. In particular, BCP-50 scaffolds, which were prepared by adding higher GelMA content in the slurry, could enhance the formation of new bone tissue and collagen matrix in the defect and have obvious osteoconductivity and osteoinductivity. These biomimetic BCP scaffolds might be the promising biomaterials to be used in bone regeneration.

## Data Availability

Data will be made available upon request.
